# Challenges in Blood Pressure Self-Measurement

**DOI:** 10.1155/2012/437350

**Published:** 2012-03-18

**Authors:** Stefan Wagner, Thomas Skjødeberg Toftegaard, Olav W. Bertelsen

**Affiliations:** ^1^Department of Engineering, Aarhus University, Dalgas Avenue 2, 8000 Aarhus, Denmark; ^2^Department of Computer Science, Aarhus University, Aabogade 34, 8200 Aarhus, Denmark

## Abstract

Blood pressure self-measurement (BPSM) requires patients to follow a range of recommendations in order to be considered reliable for diagnostic use. We investigated currently used BPSM interventions at four medical clinics combined with an online questionnaire targeting BPSM users. We found that the participating healthcare personnel perceived BPSM as a relevant and useful intervention method providing that the recommendations are followed. A total of six challenges were identified: (1) existing devices do not guarantee that the recommendations are followed, (2) healthcare providers cannot verify whether self-monitoring patients follow the recommendations, (3) patients are not aware of all recommendations and the need to follow them, (4) risk of patient induced reporting bias, (5) risk of healthcare provider induced data-transfer bias, and (6) risk of data being registered as belonging to the wrong patient. We conclude that existing BPSM interventions could be significantly affected by user-induced bias resulting in an indeterminable quality of the measurement data. Therefore, we suggest applying context-aware technological support tools to better detect and quantify user errors. This may allow us to develop solutions that could overcome or compensate for such errors in the future.

## 1. Background

Hypertension is defined as elevated blood pressure (BP) above 140 mm Hg systolic and 90 mm Hg diastolic when measured under standardized conditions [1]. Hypertension can be a separate chronic medical condition estimated to be affecting a quarter of the world's adult population [2], as well as a risk factor for other chronic and nonchronic patient groups. Traditional high-risk patient groups include diabetics, pregnant women with gestational diabetes or Preeclampsia, and kidney disease patients. For chronic hypertensive patients, persistent hypertension is one of the key risk factors for strokes, heart attacks, heart and kidney failure, and other heart and circulatory diseases and increased mortality [3]. Preeclampsia is the most common cause of maternal and fetal death [4]. For gestational diabetes and Preeclampsia patients, the accurate measurement of BP during pregnancy is one of the most important aspects of prenatal care. For kidney disease patients and diabetics, blood pressure should be kept below 130 mmHg systolic and 80 mm Hg diastolic to protect the kidneys from BP-induced damage [5].

As there are usually no symptoms, frequent blood pressure controls are highly relevant for these high-risk groups. The level of the blood pressure is the main factor in the decision to start antihypertensive therapy and other interventions. It is thus vital that the measurements are obtained in a reliable manner [6]. Measurements can be performed either at the clinic or in the home setting. In the clinical setting, patients often exhibit elevated blood pressure. It is believed that this is due to the anxiety some people experience during a visit to the clinic. This is known as the white coat effect and is reported to be affecting between 20% to 40% of all patients visiting a clinic [7, 8]. As a consequence, the current international guideline on BP measurement, is to followup on measurements obtained in the clinic using BPSM to negate the white coat effect [5–9].

BPSM is used to diagnose patients suspected of being hypertensive, as well as for long-term monitoring. BPSM is considered a valid method for determining the blood pressure (BP) of patients with hypertension and other BP-related conditions, providing that the best-practice recommendations for obtaining the measurements are followed [10–12]. These recommendations are defined by a range of national and international clinical associations [13–17].

Recommendations include the following: patient should be sufficiently rested and seated correctly before and during measurement; patient should reside in a quiet environment and should not talk during measurement. Recommendations vary between the different countries and organizations, but in general they cover the same fundamental topics.

The aim of this study is to investigate currently used BPSM interventions and identify challenges that could influence the resulting data quality. We will also discuss possible solutions to such challenges.

## 2. Methods

We planned an observational descriptive study consisting of a series of field studies at two medical clinics and two hospital departments combined with a questionnaire. Field studies were performed as a combination of observations [18, 19] of patients getting instructions and performing BPSM, combined with open-ended and semistructured interviews [19, 20] with healthcare providers, including doctors and nurses. Main objectives of the field studies were to investigate current state-of-the-art and usage of BPSM devices in the public Danish healthcare sector, including how patients are instructed and BPSM data is handled. A questionnaire targeting BPSM users was posted at the web site of the Danish Heart Foundation [21] to investigate user background and subjective understanding of the recommendations. The primary qualitative findings of the study were analyzed, thematized, and triangulated with the literature [18, 22].

### 2.1. Field Study Design: Blood Pressure Clinic

The Department of Internal Medicine and Cardiology, Aarhus University Hospital, covers a population of 200.000 people and has around. 20.000 yearly scheduled patient-contacts including hypertensive patients. Primary age groups are the middle aged and seniors [23]. As part of the department, the Blood Pressure Clinic (BPC) specializes in severe hypertensive patients referred from other departments and the primary sector. During a two-day field study, we interviewed the consultant cardiologic physician in charge of the BPC in an open-ended interview [20] on the relevance of BPSM, current clinical praxis, state-of-the-art, and other topics.

We also followed a specialist nurse while instructing hypertensive patients on how to perform both 24-hour ambulatory BPSM (ABPSM) and 3 days of home BPSM (HBPSM). We observed how the nurse conducted interviews and training with five patients to establish various aspects of the patients' overall health condition and use of medication, as well as handling of patients returning from BPSM in the home setting.

### 2.2. Field Study Design: Department of Obstetrics

The Department of Obstetrics, Aarhus University Hospital, delivers around 5.000 babies each year and performs 20.000 scans. Also, at the department, the majority of Eastern Jutland's pregnant women, covering a population of around 2.9 million suffering from complications in their pregnancy are received. In all 14.000 visits to the obstetric outpatient clinic are received yearly. This includes pregnant women suffering from hypertension, diabetes, Preeclampsia, and other complications. Some pregnant women need to visit the clinic up to 19 times during a pregnancy, for self-measuring BP, weight, protein levels, blood sugar, and fetal CTG, mainly in an unsupervised setting. Three nurses where followed in their daily routines spanning two days, first in a purely observational study observing five patients, then followed by a contextual inquiry into the routines with follow-up questions. We interviewed a physician and the department nurses on the clinical praxis of BPSM, patient training, and data capture.

### 2.3. Field Study Design: Medical Clinics

Most hypertensive patients in Denmark are treated by their general practitioner in a medical clinic. Only severe cases are referred to a specialist hospital ward such as the BPC. We visited two medical clinics in the Eastern Jutland covering around 8.000 patients in total. Both clinics are typical examples of a modern general practioner's (GP) clinic, with a community of four-to-six physicians and several support staff, including nurses and secretaries. At the two medical clinics over a period of three days, we interviewed two GP's and two nurses on the topic of HBPSM, while also discussing medical adherence and related topics.

Also, data delivery was discussed, including requirements from the staff on how data could arrive at the clinic and be sent automatically from the home of the patient and problems with staff reporting bias. We observed how BP was measured while at the clinics and how patients were counseled on HBPSM and trained to follow the recommendations. Also, three videos were recorded of nurses instructing patients on HBPSM for future reference.

## 3. Results

### 3.1. Relevance of BPSM Interventions

All the interviewed physicians and nurses reported that elevated BP levels up to 20–60 mmHg were not uncommon when measured in the clinic, as compared with patients later self-monitoring their actual BP at home confirming the daily presence of the white coat effect. Thus, BPSM is considered an important and relevant diagnostic intervention for uncovering the actual and unbiased BP of the patients by all healthcare providers involved in the study.

### 3.2. Current Clinical Praxis and State-of-the-Art

We found that all four clinics use either ABPSM devices for obtaining a 24-hour profile of patient BP with 15-minute interval, or three days of point measurements with a HBPSM device. ABPSM is used as the primary method in the BPC clinic and is considered the gold standard in all clinics. Patients who do not respond well to ABPSM are instead sent home with a HBPSM device. ABPSM is used rarely in the two GP clinics, with HBPSM being the predominant intervention. The Department of Obstetrics do not use ABPSM but rely on patients using HBPSM devices unsupervised, while attending the outpatient clinic as part of their weekly visit. BPC is the only clinic that has automatic data capture of HBPSM data, the other three relying on the patient keeping a manual paper log of measurements.

### 3.3. Importance of Following the Recommendations

We found that all the studied clinics follow the recommendations from the Danish Hypertension Association (DHA) [16] providing training for their patients on how to perform reliable HBPSM or ABPSM. With HBPSM we witnessed a total of ten patients getting instructed by nurses at the four clinics on how to perform reliable HBPSM following the established recommendations over the next three days. These instructions varied slightly in scope, but all followed the overall guidelines as defined by the DHA [16]. Also, we observed the patients performing a trial measurement. To the HBPSM patients, the healthcare providers would reiterate the importance of following the recommendations as handed out on paper, including taking the measurement on the same time of day, not lending the device to other people as the device cannot differentiate between users. Besides the recommendations from the Danish Hypertension Association, the nurse at the BPC clinic also instructed the patient not to look at the first of the three measurements in a series, in order not to build up anxiety levels.

### 3.4. HBPSM Healthcare Process

In all studied clinics we found that the HBPSM healthcare process is initiated with an interview, after which the patient is instructed in correct HBPSM usage including a supervised trial self-measurement. Next, the healthcare provider arranges for a new appointment for the patients to return with the device after three days of measurement except at the Department of Obstetrics where the patients self-measure at the clinic only. The healthcare process ends with a nurse or physician collecting the data, either manually transferring the data from the paper log or using a data cable connecting the clinic computer to the HBPSM device, before handing the dataset over to the treating physician for further diagnosis. This process is typically repeated in a half-yearly or quarterly cycle as hypertension is considered a life-long chronic condition. At the BPC clinic, the patients are referred to their GP for future reference, once they are considered well treated. Likewise, at the Department of Obstetrics the pregnant women are only followed during their pregnancy and then referred to their GP for followup.

### 3.5. Blood Pressure Devices Used for BPSM

During our interviews with GPs and cardiologists, we found that the HBPSM and ABPSM devices used by healthcare professionals are required to be on a limited list of approved devices [16, 24]. All devices we observed being used during the field studies were later confirmed to be on this list. A review of the various device-manufactures product pages revealed that none of these BP devices supported verifying patient compliance to the recommendations.

### 3.6. Trusting in Patient Ability to Correctly Self-Measure

We found that the healthcare personnel in general trusted in their patient's abilities to perform correct home measurements. However, the two interviewed GPs also stated that they had no way to ascertain the actual level of patient adherence to the recommendations. Both interviewed GP's reported that, when a patient's self-reported data indicated that the patient had done a procedural mistake, the GP would ask the patient to demonstrate a sample measurement for verification of patient understanding of the procedure. However, both interviewed GP's stated to have limited time to deal with such suspicions in daily praxis and did not keep records of the problem. As such, the level of user error could not be quantified.

### 3.7. Patient Understanding of the Recommendations

The field studies only provided limited observations useful for evaluating patient awareness and understanding of the recommendations. At the BPC clinic, one patient had taken off his ABPSM device during the night as it annoyed him. During the field studies at the obstetric outpatient clinic we witnessed five pregnant patients self-monitoring. Here, we observed user errors in four out of five measurements that could indicate limited patient understanding of the recommendations. User errors included one patient having crossed legs and back unsupported during measurement, another patient talking with the nurse during measurement, while the last two patients did not rest for the required amount of time prior to measurement.

### 3.8. Patient-Induced Reporting Bias

The field studies did not provide any direct observations of patient-induced reporting bias. However, the interviewed nurses and physicians relayed their suspicions on this occurring as BP data developments would sometimes not appear realistic. This could either be due to lack of patient understanding of proper protocol or due to reporting errors. As there are currently no means available for the healthcare personnel at the two GP clinics and the obstetric outpatient clinic to reliably detect such patient-induced reporting bias, the interviewed personnel could not account for the level of the problem. At the BPC clinic, patient-induced reporting bias is not conceivable, as data are recorded and transferred electronically.

### 3.9. Nurse-Induced Reporting Bias

At the two GP clinics, both nurses and GP's stated that measurements could be subject to nurse-induced reporting bias when entering the individual measurements into the GP electronic patient record system. Several examples were given including difficulties in reading the patient's hand-written diaries, transition from paper to screen errors, calculation errors while getting the average BP levels, and other sources of bias. It was not possible to quantify the rate or severity of errors, but physicians and nurses from the two GP clinics deemed the problem to be relatively frequent.

### 3.10. Risk of Wrong Patient Measurements

The personnel at the BPC clinic suspected that some of the BPSM measurements that would automatically be recorded and transferred to the clinical computer system might stem from other persons than the intended patients. As patients could easily lend out their devices to other persons, for example, relatives or friends, either knowingly or unknowingly, there was a potential risk of unintended persons' BP measurements ending up as part of the originally intended patients BP data set.

At the two GP clinics and the obstetric outpatient clinic, the employed BPSM procedure relies on patients self-reporting data, transferring them manually from device screen to paper. Here, this did not appear to be an issue due to the manual nature of the procedure.

### 3.11. Findings from the Questionnaire Study

**Figure 1:**
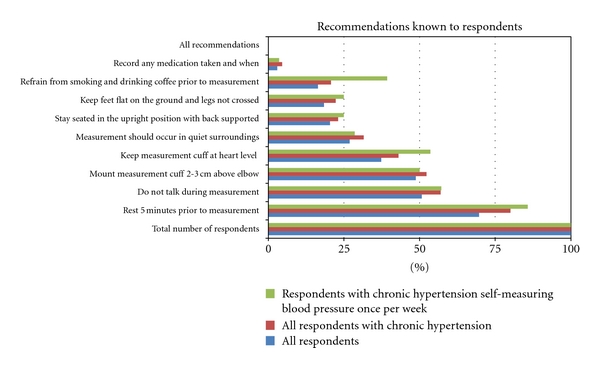
Illustrates the percentage of respondent understanding and knowledge of the recommendations when performing self-measurement of blood pressure. A total of 201 respondents provided answers as indicated in the subgroup color-coded blue. Of these, some 130 respondents self-reported to suffer from chronic hypertension as indicated in the second subgroup (red). In the third subgroup (green) a total of 28 respondents self-reported to be suffering from chronic hypertension as well as self-measuring their blood pressure once per week. The percentage-figures are relative to each of the three subgroups.

A questionnaire was posted at the website of the Danish Heart Foundation advertising for participants using HBPSM. A total of 201 respondents in the age groups 20–80 provided answers. Of these, 130 (65%) self-reported to suffer from hypertension and 124 (62%) reported taking hypertensive medication. Other respondents included hypotensive patients, normotensive, and diabetics. A total of 124 (62%) reported having a BP measurement device in their home. More than half of the respondents, 121, measure their BP at least once every third month at home, 35 (17%) once a week, 24 (14%) biyearly, 6 (3%) yearly, while 24 (12%) do not self-measure but get their BP measured at the clinic. Only 3 (1.5%) respondents reported not measuring their BP at all. When asked about knowledge of the selected recommendations, 140 (70%) of respondents knew that they had to rest five minutes prior to measurement. The recommendation not to talk during measurement was known by half of the respondents. Another 86 (43%) knew both of these recommendations, while none of the respondents had knowledge of all of the recommendations. See Figure 1 for an overview of respondent's detailed knowledge of the recommendations.

Focusing the result set to those 130 respondents who self-reported to suffer from chronic hypertension, a total of 104 (80%) of respondents knew that they had to rest five minutes prior to measurement. The recommendation not to talk during measurement was known by 74 (57%) of the respondents. Another 65 (50%) knew both of these recommendations, while none of the self-reported hypertensive respondents had knowledge of all of the recommendations.

Further narrowing the scope to those measuring their BP once a week limits the number of respondents to 28. The majority of these, 24 (86%), knew that they had to rest 5 minutes prior to measurement, while 57% knew that they should not talk during measurement. See Figure 1 for further details.

## 4. Discussion

### 4.1. Using HBPSM Following the Recommendations

From both the field studies and the literature, it appears that HBPSM interventions are considered useful for avoiding white-coat-induced bias and obtaining the actual blood pressure of the patients [8, 12, 14, 25, 26]. Results indicate that the studied nurses and doctors all recognize the importance of following the recommendations for HBPSM interventions in the unsupervised setting and that they are instructing their patients on how to perform correct measurements in accordance with this.

However, several potential challenges that could affect the data quality of HBPSM measurements stand out from the study findings. In the following we provide a list of such potential challenges triangulated with findings from the literature.

### 4.2. Challenge 1: Existing Devices Do Not Guarantee That the Recommendations Are Followed

Findings from the field studies indicate that existing state-of-the-art BP devices do not automatically ensure that the recommendations are followed. Some devices are able to store BP readings along with date and time meta-data. Such devices have previously been used to determine the level of compliance with reporting self-measured data [27–30]. However, the majorities of clinics visited (75%) does not use the storage features of the BP devices but rather relies on paper-based log books for data capture and exchange. Furthermore, in the biomedical BP devices that are currently being used in the studied clinics, it is only date-and-time contextual meta-data that is captured and not factors related to the remaining recommendations. A survey of all BHS-approved devices indicate that no state-of-the-art devices are available that can ensure that any of the recommendations as presented in Figure 1 are followed [16, 24].

### 4.3. Challenge 2: Healthcare Providers Cannot Verify Whether Self-Monitoring Patients Follow the Recommendations

As observed in the field studies, the healthcare providers relied on the individual patient's ability to perform a correct measurement in the unsupervised setting, either the home or the outpatient clinic, being dependent on the patients training and willingness to adhere to the recommendations. Healthcare providers were not able to quantify adherence levels of the patients, beside time and date of measurements, and the BP measurements themselves.

### 4.4. Challenge 3: Patients Are Not Aware of All Recommendations and the Need to Follow Them

Patients are instructed by the healthcare professionals to use the home BP device for self-measurement, either in the home setting or at the outpatient clinic, and to follow the recommendations provided by the healthcare professionals. Findings from the field studies and the questionnaire study indicate that patients are not always aware of the recommendations and the importance of following them. As a consequence patients may only be following them in part or not at all. The questionnaire study indicated that none of the respondents were aware of all the recommendations, while 91% were aware of one or more. This was further supported by our observations in the field study, where we observed users not following the recommendations during self-measurements at the obstetric outpatient clinic.

### 4.5. Challenge 4: Risk of Patient-Induced Reporting Bias

Patients who are keeping manual log of their BP self-measurement data could risk introducing errors during the transfer of data from the device display to the paper log. This could occur not only through rounding errors or misreading of digits but also through accidental or deliberate deletion of individual measurements. Findings from the field studies indicate that not all healthcare professionals, including the staff at Aarhus University Hospital, Skejby and at the two GP clinics, use automated data transfer from the biomedical devices. As 75% of the studied clinics rely on manual paper logs, this could indicate a potential risk of patient-induced reporting bias. Previous studies have found reporting bias to affect up to 50% of all BP self-measurements [27–30]. Reporting bias, both healthcare provider and patient induced, is however easy to avoid by using state-of-the-art equipment with the ability to transfer data automatically from a BP device to electronic healthcare records. This was only used in one of four clinics in the study.

### 4.6. Challenge 5: Risk of Healthcare-Provider-Induced Data-Transfer Bias

Findings from the field studies indicate, that patients keeping manual logbook of their self-measurement of BP data require the nurse (or other healthcare provider) to transfer data from the logbook to the computer-based records. In this process the healthcare provider could risk inducing errors during the transfer. In the literature, this type of error has not been investigated thoroughly, and the level of data-transfer bias has not been quantified in the field studies, though it was indicated at both GP clinics as a potential risk. 

### 4.7. Challenge 6: Risk of Wrong User Data Being Registered

In the case of another user than the intended patient using an automatic BP device for performing a measurement, the data will automatically, but erroneously, become part of the data set of the patient it is registered to. The existing and currently employed automatic BP devices have no means of differentiating between different users, and the data cannot be easily removed from the system by the user [16, 24]. This risk was explicitly pointed out at the BPC clinic to patients during their training sessions. In the three other clinics, this was not viewed as a likely problem, as the patient keeps a manual paper log that most likely would mitigate this challenge.

### 4.8. Clinical Implications

Results indicate that BPSM as a clinical method could be subject to bias to such an extent that results are unfit for diagnostic, monitoring, or scientific use. A user talking during self-measurement could bias the measured BP data levels with as much as 7–20 mm Hg [6, 13], which would suffice to change patient diagnosis from normotensive (healthy) to hypertensive, thus requiring medication [10]. Other bias could be introduced from other activities while underreporting could have the opposite effect for some. In the literature, underreporting has been reported to occur frequently and on a significant scale [28–30]. This could affect the data quality of previous clinical studies, such as those reviewed by AbuDagga et al. [31] where study methods rely on such unsupervised self-measured data sets.

Furthermore, unsupervised healthcare self-measurement techniques could even be perceived as misleading or harmful, resulting in misdiagnosis and potential over- or under-medication of patients [10]. However, as observed in the field study and known from the literature, the prevalence of the white coat effect necessitates HBPSM in the home of the patient as it is the only method to provide the actual BP of patients suffering from this syndrome [8]. As such, BPSM in the unsupervised setting is likely to remain a commonly used clinical method. Other incentives for utilizing BPSM include improved data material through more frequent measurements as well as for the convenience of the patients as they do not need to visit the clinic for having their measurements taken [30, 31].

The findings from the four reported field studies indicate that the problems of potentially reduced measurement-adherence reported in the literature could be relevant as it cannot be verified whether patients self-measuring at home are always following the recommendations as intended. A patient not following the recommendations in full leads to biased data and may thus result in potential misdiagnosis of patients. The extent of the problem has not been quantified, as this requires further studies. However, previous work has indicated that reporting bias alone may be affecting a large proportion of all self-measurements (up to 50%) and that this could be a major problem [28–30]. As reporting bias primarily consists of reading digits on the display and transferring them to paper, other problems which cannot be measured with existing technology could be a much greater problem.

### 4.9. Implications for Future Work

We have identified a range of potential challenges related to BPSM in the unsupervised setting. However, our findings are based on a limited qualitative empirical data material and should be treated as indicative. In order to investigate these challenges further, we need methods and tools that will allow us to gain a better understanding of how the patient is acting in situ in the home setting or at the outpatient clinic while self-monitoring. Traditional methods useful for this kind of research activities include observations in the home setting by researchers, either directly or through video capture [19, 20]. This requires the researchers to be present in the home and may be highly time consuming and privacy invading. As such, this method is not feasible for anything but limited qualitative studies. There is also the risk of inducing bias on patient behavior during measurements, while being observed. The presence of researchers might prompt patients to follow recommendations to a larger extent when being observed. Video recordings are less intrusive and can even be undertaken with hidden camera installations. However, they still require a fairly large technical setup as well as extensive reviewing work. Also, the ability to correctly capture all context information might also be limited when using a single camera only. It might for example be difficult to capture video for verifying both whether the user is correctly seated while also monitoring placement of the feet and the handling of the BP device.

As an alternative to direct or indirect visual observations, we suggest that it may be feasible to develop research prototypes that can discretely measure the desired use-context during a healthcare session. For example, we may be able to develop a system with sensors to detect whether the user is correctly seated and measure noise levels during BPSM to quantify adherence levels. Rather than by direct observations or video reviews, the researchers will gain a formalized and discrete data set of relevant user behavior that can automatically be data-processed and correlated with the measurements. In this way, it may arguably be faster to perform longitudinal studies on a larger population, leading to a more comprehensive understanding than could be achieved with manual observations and data processing. Creating such systems is however a nontrivial task. Depending on the problem under investigation, there is a need for a data acquisition platform, consisting of a computer with relevant processing capabilities, sensors for data capture, a sufficient infrastructure, relevant user interface hardware, video and audio recording, patient identification, and other relevant features. We find that context-aware technologies and concepts appear promising to meet these requirements. Context-aware technology is an established area within the combined fields of ubiquitous and pervasive computing [32–34]. Dey has previously introduced the concept of “context-tagging of information” [35], where pervasive systems have the ability to attach relevant context meta-data to the primary data set. Applying these concepts to the identified challenges of this study appears relevant. This could include attaching relevant context information to a BP measurement data set, such as whether the user was sufficiently rested when performing the measurement, seated correctly when medication was taken, as well as any other relevant contextual factors which are known to impact BP measurements.

To this purpose, a range of context-aware and extendable middleware technologies intended for developing context-aware pervasive healthcare [36, 37] and telemedicine prototypes are available. This includes SOPRANO, OpenAAL, JCAF, AMICA, Hydra, SPINE, and OpenCare [38–43]. Building a platform for evaluating the reliability of BPSM and other relevant telemedicine topics by acquiring relevant context information using this type of framework appears feasible and relevant. We suggest developing systems using existing context-aware technologies, for learning more on user behavior in the unsupervised setting.

An improved understanding of the disease domain and the related challenges could also be used to suggest novel strategies based on technological solutions for improving measurement-compliance. Through the of use context-aware technology, we may be able to better quantify the level of compliance with the recommendations, thus providing a marker on the quality of the data obtained in the home setting or during self-measurement in general.

Lessons learned from experimental research prototypes may also contribute to designing more advanced biomedical devices for clinical use enabling BP devices to better sense the measurement context and possibly provide better user guidance relying on context-aware technologies [35]. This could include sensing noise levels and user activity levels, as well as whether the user is correctly seated. All this is feasible with current context sensor technology. If all bias sources and their relative effect on BP can be sufficiently determined, then it might be possible to automatically compensate and filter out such bias of the resulting BP, providing a more valid data set to the healthcare provider.

Findings could also be applied to state-of-the-art pervasive healthcare telemedicine solutions, such as the Intel Health Guide PSH6000 [44] and the Tunstall mymedic TeleHealth Monitor [45]. These healthcare platforms have been used in several clinical trials, including in the TELEKAT project [46]. Refitting such systems with context-aware technology for sensing user behavior is possible through the Continua Alliance open standards [47] and would allow us to quantify bias during daily use. This class of systems also provides enhanced communication features for easier user guidance, as well as more processing power than current stand-alone biomedical devices feature today. Finally, results could be useful for inspiring future smart home infrastructure technology design [48, 49]. Here, a built-in infrastructure for context awareness and user interaction is contemplated as being an integral element of every future smart home allowing us to track noncompliant user behavior [50, 51].

## 5. Conclusion

We found that the studied healthcare personnel perceived BPSM as a relevant and useful method for obtaining the unbiased BP of patients, providing that the clinical recommendations are followed. Contrasting this, the questionnaire respondents appeared to have an incomplete understanding of the recommendations and the need to follow them during BPSM. We identified six challenges related to using the BPSM method. These challenges indicated frequent and severe user-induced bias during BPSM which could result in an indeterminable data quality of the measurements. Biased and unreliable data leads to potential misdiagnosis of patients, possibly affecting large patient groups in current clinical praxis. Also, the challenges and resulting bias may impact the validity of results from previous clinical trials on BPSM, as patient compliance with the guidelines has not been investigated sufficiently in these studies. In order to gain a better understanding of the six challenges and their consequences, we have proposed developing novel methods and context-aware technological support tools to better detect and quantify such user errors. These tools could also allow us to develop and investigate experimental assistive measures to help users overcome the challenges themselves or automatically compensate for any measurement bias. We suggest that context-aware methodology and technology could be useful for such purposes building on the existing body of work within the pervasive healthcare community, including software frameworks and sensor technology. In the future our research could be used to develop better biomedical BP devices, as well as improve existing telemedicine and telemonitoring platforms and inspire future smart home technology to overcome the six identified challenges.
